# Physical Mapping of Peroxidase Genes and Development of Functional Markers for *TaPod-D1* on Bread Wheat Chromosome 7D

**DOI:** 10.3389/fpls.2019.00523

**Published:** 2019-04-24

**Authors:** Hongwei Geng, Jia Shi, E. Patrick Fuerst, Jingxin Wei, Craig F. Morris

**Affiliations:** ^1^College of Agronomy, Xinjiang Agricultural University, Ürümqi, China; ^2^Department of Crop and Soil Sciences, Washington State University, Pullman, WA, United States, Affiliated with the Western Wheat Quality Laboratory; ^3^National Wheat Improvement Center, Institute of Crop Science, Chinese Academy of Agricultural Sciences (CAAS), Beijing, China; ^4^USDA-ARS Western Wheat Quality Laboratory, E-202 Food Science and Human Nutrition Facility East, Washington State University, Pullman, WA, United States

**Keywords:** *Triticum aestivum* L., peroxidase, gene cloning, physical mapping, gene-specific markers

## Abstract

Peroxidase (POD) activity in wheat (*Triticum aestivum* L.) grain influences natural carotenoid pigment content and is associated with the color of flour, and processing and product quality. Here, we report the molecular characterization and physical mapping of POD genes in bread wheat. The complete genomic DNA (gDNA) sequence of two POD genes (*TaPod-A2* and *TaPod-D1*), and the partial gDNA sequence of two additional POD genes (*TaPod-A3* and *TaPod-B1*) from wheat were characterized using *in silico* cloning and validated through laboratory experiments. Using a set of 21 nullisomic-tetrasomic (NT) lines, six group-7 ditelosomic (Dt) lines, and 38 group-7 deletion (Del) lines of Chinese Spring (CS), *TaPod-A2* and *TaPod-D1* were found to be physically located on 0.73–0.83 and on the most distal 0.39 fraction arm length (FL) of 7AS and 7DS in cv. CS, respectively; whereas, *TaPod-A3* and *TaPod-B1* were assigned to the 0.40–0.49 and 0.40–0.48 FL of 7AL and 7BL, respectively. Based on single nucleotide polymorphisms (SNPs) of two alleles at the *TaPod-D1* locus, two functional markers POD-7D1 and POD-7D6 were developed, amplifying 540- and 640-bp, fragments in varieties with higher and lower POD activities, respectively. A total of 224 wheat varieties were analyzed and showed a significant association between the polymorphic fragments and POD activity using POD-7D1 and POD-7D6 markers. The analysis of variance (ANOVA) indicated the average POD activities of 115 varieties with *TaPod*-*D1a* were significantly lower than 109 varieties with *TaPod*-*D1b* (*P* < 0.01). This study provides useful information of the POD genes in bread wheat, insight into wheat genome synteny and structure, gene-specific markers, and contributes a valuable resource for quality improvement in wheat breeding programs.

## Introduction

Peroxidases (PODs) are heme-containing oxidoreductases, ubiquitous in plants and animals, and can oxidize various hydrogen donors at the expense of hydrogen peroxides (H_2_O_2_) (Hiraga et al., 2001; Jouili et al., 2011). Traditionally, the plant POD superfamily was classified into three types according to differences in primary structure and catalytic properties and denoted using Roman numerals: I, II, and III. Among higher plants, two POD classes are recognized: class I PODs (ascorbate POD, EC1.11.1.11), which are intracellular, and class III PODs (guaiacol POD, EC1.11.1.7), which are secreted into the vacuole, the cell wall, and the surrounding medium (Fraignier et al., 2000). The class III plant PODs (hydrogen donor: H_2_O_2_ oxidoreductase) are classical PODs of higher plants and have an affinity for guaiacol, which is catalyzed by the reduction of hydrogen PODs by taking electrons from hydrogen donor molecules (Hiraga et al., 2001; Delannoy et al., 2006; Csiszár et al., 2008; Shigeto and Tsutsumi, 2016). Previous studies proposed that PODs are not specific in their reactions, and can oxidize various phenols and aromatic amines of plant tissues by H_2_O_2_ (Gelinas et al., 1998; Feillet et al., 2000; Zhai et al., 2013). Hence, the plant PODs are involved in various physiological functions such as removal of hydrogen peroxides, senescence, oxidation of toxic reductants, lignin formation, pathogen defense, or insect attack (Yoshida et al., 2003; Maksimov et al., 2010).

Peroxidases are widely distributed in cereals (Zhai et al., 2013; Shigeto and Tsutsumi, 2016). Previous studies identified POD as being responsible for pigment degradation, especially ß-carotene and lutein oxidation (Iori et al., 1995; Fraignier et al., 2000). An abundance of hydrogen peroxide is produced during intermediate steps of substrate peroxidation in the POD pathway (Yoshida et al., 2003; Maksimov et al., 2010). This hydrogen peroxide can oxidize various natural carotenoid pigments such as ß-carotene and lutein in seeds; reduce the color of flour and ultimately deteriorate the quality of wheat-based end products (Kobrehel et al., 1974; Delcros et al., 1998). Conversely, POD can improve the rheological properties of flour doughs and the physical characteristics of gluten by oxidizing sulfhydryl (-SH) groups of cysteine to form intra- and intermolecular disulfide bonds, and can improve rheological properties of dough in bread making technology (Gelinas et al., 1998; Figueroa-Espinoza et al., 1999; Borrelli et al., 2003; Revanappa et al., 2014). POD action exerts significant effects on deteriorative changes in flavor, texture, nutritional value, and mouth feel of food products for human consumption (Burnette, 1977; Takasaki et al., 2005). Furthermore, POD activity can induce the development of undesirable pasta brownness (Taha and Sagi, 1987; Fraignier et al., 2000). A significant positive correlation (*r* = 0.84–0.97) was observed between the brown index of pasta products and POD activity (Iori et al., 1995; Hemalatha et al., 2007). Similarly, a high positive correlation (*r* = 0.81) was reported between darkening of chapattis and POD activity (Hemalatha et al., 2007). Furthermore, POD supplementation bleaches dough and increases dough tolerance to over-mixing (Hilhorst et al., 1999; Joye et al., 2009). POD seems to drive the formation of dityrosine bonds during bread making and can improve loaf volume and crumb structure (Joye et al., 2009). Particularly in soft wheat, POD is involved in arabinoxylan and protein cross-linking, which affects batter viscosity and processing quality (Bettge and Morris, 2007; Ramseyer et al., 2011a,b).

Feillet et al. (2000) showed that POD activity was influenced by genotype, environment, and genotype × environment. However, Kobrehel and Gautier (1974) suggested that POD is a genetic character, where different genomes control the synthesis of specific PODs in wheat, and environmental factors had a minor effect on the isozyme patterns. Common wheat showed the highest POD activity among the cereals and was three, six, and seven times higher than oat, rice, and maize, respectively (Maksimov et al., 2010). Žilić et al. (2010) reported that bread wheat showed a higher POD activity than durum wheat. In addition, a wide range of intervarietal variation has been reported between wheat varieties for POD activities (Honold and Stahmann, 1968; Borrelli et al., 2003; Hemalatha et al., 2007; Maksimov et al., 2010). These studies have shown that it is possible to change POD activity of wheat by genetic selection. By analyzing NT lines of cv. CS wheat by polyacrylamide-slab gel electrophoresis, Kobrehel and Feillet (1975) identified six POD isozymes (denoted a, b, c, d, e, and f) in bread wheat endosperm. Using NT lines of CS, POD a, c, and d were located on 7D, 4B, and 7A, respectively, whereas isozymes e and f appeared to be located on at least two different chromosomes. The POD isozymes showed a higher tissue-specificity but a lower substrate-specificity, and the embryo plus scutellum and the endosperm of durum wheat always presented different POD patterns (Fraignier et al., 2000). Bosch et al. (1987) also found that the embryo plus scutellum PODs were associated with 3AL, 3BL, and 3DS; whereas wheat endosperm PODs were associated with 4BL, 7AS, and 7DS. Recently, three major QTLs associated with POD activity (*QPod.caas*-*3AL*, *QPod.caas*-*4BS*, and *QPod.caas*-*5AS*) were detected and associated with a wide range of phenotypic variation (5.3–21.2%) across four environments using a recombinant inbred line (RIL) population (Doumai/Shi 4185) and the wheat 90 K single nucleotide polymorphism (SNP)-chip markers (Wei et al., 2015).

To date, several plant POD genes were cloned in rice (Passardi et al., 2004a,b), maize (Mika et al., 2008), barley (Johansson et al., 1992; Theilade and Rasmussen, 1992), wheat (Wei et al., 2015), and *Arabidopsis thaliana* (Tognolli et al., 2002). Plant PODs exist in numerous molecular forms. More than 138, 73, and 200 different sequences encoding POD genes have been identified in rice (Passardi et al., 2004a,b), *A. thaliana* (Tognolli et al., 2002), and maize (Mika et al., 2008), respectively. The barley PODs are coded for by more than four loci: one (seed POD) on chromosome 1, two on chromosome 2, and one on chromosome 5 (Johansson et al., 1992). A barley POD, *Prx 5* (mRNA, GenBank accession number M73234), has been isolated from mature barley grains, and was RFLP-mapped on barley chromosome 3. Analyses of RNA and protein (Prx 5) were found only in the endosperm, and not expressed in leaves (Rasmussen et al., 1991). Theilade and Rasmussen (1992) purified a barley genomic DNA (gDNA) sequence (*Prx 6.1*, M83671) using a complementary DNA (cDNA) coding for POD in the barley seed, and *Prx 6.1* was RFLP-mapped on chromosome 3. The other barley POD gene, *Prx 6.2* (Z23131, unpublished) was located on chromosome 3.

Ten full-length cDNA encoding POD, *TmPRX1* to *TmPRX10* (GenBank accession numbers AY857755 to AY857764), were cloned from leaf epidermis of diploid wheat (Liu et al., 2005). These 10 POD genes are diverse and encode proteins of 312–357 amino acids (AAs) with a signal peptide based on bioinformatic analysis (Liu et al., 2005). Two common wheat cDNA sequences (AF525425 and AK331797) were found by a BLAST search using the rice gDNA sequence BN000552 as a probe. Wheat cDNA AF525425 and AK331797 showed 73.8 and 73.3% sequence identities with BN000552, respectively. The cDNA of *Wheat* POD*-1* (*WP1*, HM138375) was amplified from immature seeds by RT-PCR (Shan et al., 2009). The cDNA of *WP1* codes a protein of 358 AA, showing 100 and 89% sequence identity with AF525425 and barley POD 1 (*BP1*, M73234), respectively. Wei et al. (2015) cloned and characterized a complete genomic wheat POD DNA sequence of *TaPod-A1*, which is mapped on 3AL. Additionally, based on the SNPs of two alleles (*TaPod-A1a* and *TaPod-A1b*), two functional markers (POD-3A1 and POD-3A2) were developed, and were highly related to POD activity (Wei et al., 2015).

There is relatively little knowledge on the POD gDNA sequences in hexaploid wheat (*T. aestivum*). Further, the physical location of POD genes has not been identified further than the whole chromosome arm. Several wheat POD genes have been cloned and two complementary dominant sequence-tagged site (STS) markers were developed for QTL controlling POD activity. However, common wheat contains a large POD gene family, thus requiring that more POD genes be precisely localized so as to manipulate the POD activities and improve the end-use quality of wheat.

Hence, the objectives of the present study were threefold. The first objective was to clone POD genes by the method of *in silico* cloning in combination with PCR amplification based on two wheat full length cDNA sequences, AF525425 and AK331797. The second objective was to physically map the POD genes, using CS ditelosomic (Dt), NT, and the Del lines of Endo and Gill (1996). The third objective was to identify allelic variants on chromosome 7D and develop functional markers that can be used to characterize wheat varieties for POD activity based on allelic variants.

## Materials and Methods

### Plant Materials

The wheat variety Alpowa (PI 566596) was used for sequencing new POD genes. Alpowa is mainly a pastry wheat (soft white spring); however, its flour can be made into all types of soft wheat products, such as crackers and flatbreads.

For mapping EST-specific fragments, new POD genes, and gene-specific markers to individual chromosomes and arms, a set of Chinese Spring (CS) nullisomic-tetrasomic (NT) lines, and six group-7 CS Dt lines were used. Thirty-eight homozygous CS deletion (Del) lines for group-7 were used for sub-arm mapping and included 10 Del lines of chromosome 7AS (CS Del7AS), 16 Del lines of chromosome 7AL (CS Del7AL), nine Del lines of chromosome 7BL (CS Del7BL), and three Del lines of the short arm of chromosome 7D (CS Del7DS). Seed of these genetic stocks was acquired from Jon Raupp (Kansas State University) and were planted in the Plant Growth facility of Washington State University, Pullman, WA, United States. The bread wheat chromosome bin maps can be viewed at the group-7 long-arm and short-arm Dt lines in CS wheat^1^.

### Field Trials and POD Activity Assay

For validation of gene-specific markers of the POD genes, during the 2012–2013 and 2013–2014 cropping seasons, 147 varieties from the Yellow and Huai Winter Wheat Region of China (YHRVWWR) were sown at Suixi in Anhui province and Anyang in Henan province, and 77 varieties of the Northern China Plain Winter Wheat Region (NWWR) were sown in Shijiazhuang and Beijing. All the field trials were conducted in randomized complete blocks with three replicates. Grains from two replicates were used for POD activity assay. Each plot comprised three 2-m rows spaced 20 cm apart. Wheat grain POD activity was assayed following Wei et al. (2015).

Peroxidase activity in wheat grain was tested by measuring increased absorbance at 470 nm using an Absorbance Microplate Reader (Molecular Devices SpectraMax 384 Plus, LLC, United States). Each sample was tested in duplicate. If the coefficient of variation (CV) was >10% for spectrophotometric assay of POD activity in the duplicates, a third test was conducted. For each data point, POD activity in wheat grain was tested in duplicate extracts of whole-wheat flour with parallel spectrophotometric measurements and average values are reported (Wei et al., 2015).

### Strategies for Cloning and PCR Parameters

The corresponding gDNA sequence of rice POD gene *PRX23* (GenBank accession BN000552) was used for a BLAST search against the wheat expressed sequence tag (EST) database in GenBank and Computational Biology and Functional Genomics (CBFG) Laboratory^2^. All ESTs in wheat sharing higher similarity with the reference gene (score > 390 bp, E value < 10–50, and identity > 85%) were subjected to overlapping sequence assembly. Six gene-specific primer sets, P1, P2, P3, P4, P5, and P6, were used to clone the whole coding sequence of POD genes. Primers were designed using the software Primer Premier Version 5.0^3^. Each primer set was detected with a set of NT lines to confirm a chromosome specific location. gDNA was extracted from leaf tissue of individual seedlings about 2–3 weeks post emergence using a method modified from Riede and Anderson (1996).

PCR reactions were carried out in a total volume of 20 μL, including 250 μM of each deoxyribonucleotide triphosphate, 100 ng of gDNA, 8 pmol of each primer, 1x reaction buffer (10 mM Tris-Cl, 50 mM KCl, and 1.5 μM MgCl_2_, pH 8.5), 1 U *Taq* DNA polymerase (5 Primer Inc., Gaithersburg, MD, United States) (Geng et al., 2012). Temperature conditions for PCR were 94°C for 5 min, followed by 40 cycles of 94°C for 50 s, 62–64°C (Table 1) for 55 s and 72°C for 1 min, and a final 8 min elongation at 72°C. Amplified PCR fragments were visualized on 1–1.5% agarose gels.

**Table 1:** The sequences of the primer sets used in the cloning of the POD genes along with their PCR profiles, product sizes.

	Primer set	Primer (5′–3′)	Amplified region	Size of PCR fragment (bp)	PCR annealing/ temperature
*TaPod-D1*	P1	Forward: AGCACACAAGGAGAGAGGAG	-20-468	488	64°C
		Reverse: AAGAGGCACGCGGTAGTCG			
	P2	Forward: CGACTACCGCGTGCCTCTT	450-1104	655	62°C
		Reverse: TAGTCCACTTGTCTAGATGCTT			
*TaPod-A2*	P3	Forward: GCTACCCTTGAATCCTGCCTA	-92-660	752	64°C
		Reverse: CTCGAAGGAGGAGCAGTGC			
	P4	Forward: GGACTACCGCGTGCCTCTC	462-1090	629	64°C
		Reverse: GCTAGCCAAGGCTTTCTTCG			
*TaPod-A3*	P5	Forward: CCTCGTCAGCGGGGTTCG		574	62°C
		Reverse: CCGTGCATTCGCATTCAAG			
*TaPod-B1*	P6	Forward: GCCTCGTCAGCGGGTTCC		580	62°C
		Reverse: CGTGCCAACACAACACACTG			
Gene-specific markers	POD-7D6	Forward: TGGGCATGGGGCTTCTGCA		640	58°C
		Reverse: GCGAGGAATGGGGGGTTGATG			
	POD-7D1	Forward: GCTTCGTCCAGGACGCCGTT		540	61°C
		Reverse: CGAGGAATGGGGGGTTGATG			


### DNA Sequencing and Analysis

Amplified PCR products were purified by ExoSAP-IT according to the manufacturer’s instructions. Sequencing was conducted with an Applied Biosystems 3100 Genetic Analyzer (PerkinElmer Applied Biosystems Division, Foster City, CA, United States), and sequencing reactions were carried out with the Big Dye Terminator Version 3.1 Cycle Sequencing Kit.

The wheat variety Alpowa was used for cloning the gDNA sequence of *POD* genes. Four varieties with a broad range of POD activities, Zhong 892, Zhou 8425B, Shi 4185, and Zhoumai 16, were used to identify allelic variants of the *TaPod-D1* gene on chromosome 7D (Wei et al., 2015). Each primer set was verified to be chromosome-specific with CS NT lines. The sequence of gDNA and deduced AA for POD genes were compared with the software DNAMAN Version 7.0 (Lynnon Corporation, 2013^4^). The AA sequences of cereal POD genes were subjected to the Conserved Domain Database^5^ for conserved domain analysis.

### Sequence-Tagged Site (STS) Analysis

Using the newly developed gene-specific markers, six wheat varieties with higher and six with lower POD activities were amplified and sequenced. Two primer combinations were designed to detect variants among these varieties using the sequence divergence of the two alleles of the *TaPod-D1* gene. The variation in POD activity was assumed to be associated with *TaPod-D1*, which was then verified on 224 Chinese wheat varieties. The PCR amplification conditions were 1 cycle of 94°C for 5 min, followed by 40 cycles of 94°C for 50 s, 58°C or 61°C (specific annealing temperature of each primer set is shown in Table 1) for 50 s 72°C for 55 s, and a final 8 min elongation at 72°C.

### Statistical Analyses

Analysis of variance (ANOVA) was conducted using SAS version 9.2 (SAS Institute, Cary, NC, United States). For the 224 Chinese wheat varieties, the POD activity of each variety was determined in each of four environments and averaged to confirm the association between POD activities and allelic variants. The variation in POD activity among varieties with different PCR fragment profiles was analyzed using Fischer’s least significant difference (LSD).

## Results

### Characterization of Complete DNA Sequence of *TaPod-D1* and *TaPod-A2*

Four common wheat cDNA sequences (AF525425, HM138375, AB518867, and AK331797) and one *Triticum monococcum* cDNA sequences (AY857762) were found through a BLAST search against the GenBank wheat EST database using the rice gDNA sequence BN000552 as a probe. AF525425, named *WSP1*, shared 100, 100, 93, and 54% sequence identify with HM138375, AB518867, AK331797, and AY857762, respectively (data not shown). Two *T. monococcum* ESTs (TC389044 and TC383617) were detected through a BLAST search against the CBFG Laboratory wheat EST database^6^. using the *T. monococcum* sequence of AY857762 as a probe. AY857762 shared 92 and 87% of sequence identity with TC389044 and TC383617, respectively.

**FIGURE 1:**
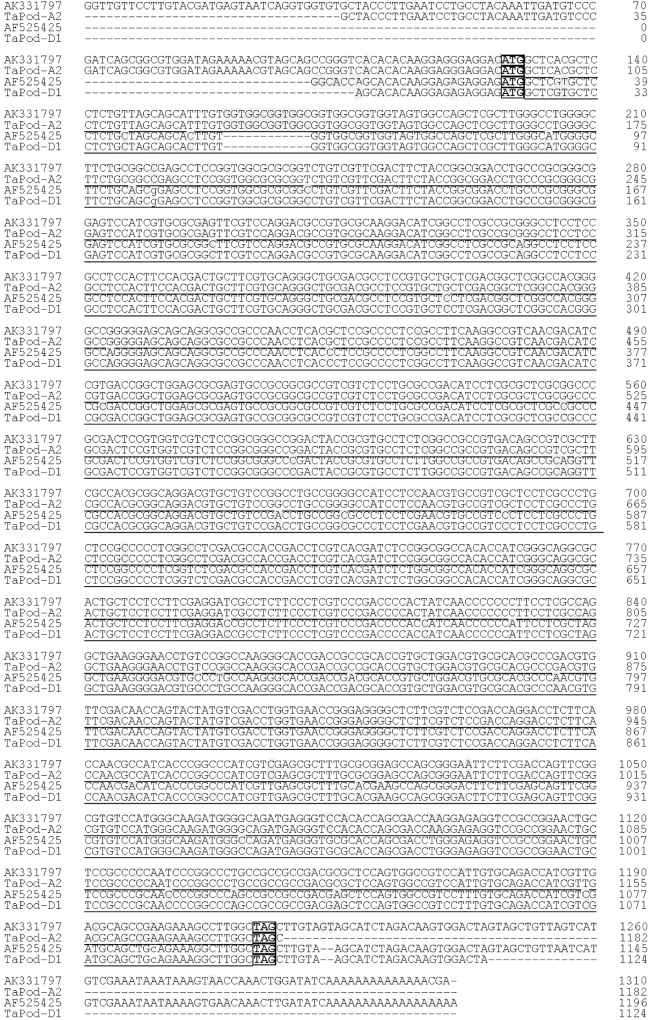
Alignment of *TaPod-D1* and *TaPod-A2*. The SNPs are *shaded*, whereas the exons are *underlined*. The start and terminate codons are in *boxes. Hyphens* indicate gaps to improve alignment.

**FIGURE 2:**
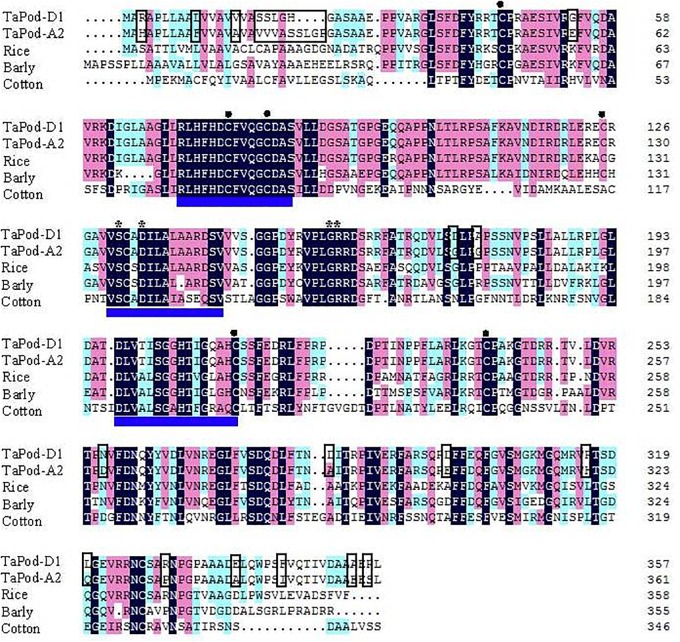
Amino acid sequence alignment of wheat *TaPod-A2* and *TaPod-D1*, rice (CAH69265), barley (AAA32974), and cotton (AAL92037). The shadows show the same amino acid residues. The different amino acids in the peptide sequences encoded by *TaPod-A2* and *TaPod-D1* are in boxes. The three conserved domains of class III peroxidase are *underlined*. The residues involved in a salt bridge are indicated with *asterisks* (^∗^). The cysteines involved in disulfide bridges are indicated with *solid circles* (^•^) (Hiraga et al., 2001; Passardi et al., 2004a,b; Marchler-Bauer et al., 2009).

Based on the SNPs and InDels among AF525425, AK331797, TC389044, and TC383617, six chromosome-specific primer combinations (designated P1, P2, P3, P4, P5, and P6) were developed (Table 1). Of them, P1 and P2 could be used to amplify the gDNA sequence of *TaPod-D1*, and were mapped on wheat chromosome 7D as identified using a set of NT lines (Supplementary Figures S1a,b). The upstream sequence of gDNA sequence of *TaPod-D1* amplified by P1 demonstrated 100% identity with AF525425, while the downstream sequence of gDNA sequence of *TaPod-D1* amplified by P2 demonstrated 100% identity with AF525425 (Table 1 and Figure 1). P1 and P2 produced PCR products of 488 and 655 bp, respectively. There were 19-bp overlaps between the PCR products produced by P1 and P2. The DNA sequence of *TaPod-D1* was comprised 1124 bp, with an open reading frame (ORF) of 1077 bp, a 5′ untranslated region (UTR) of 20 bp, and a 3′ UTR of 27 bp. The predicted pre-protein sequence of *TaPod-D1* consisted of 358 AAs, with a predicted molecular weight of ∼38.8 kDa (Figure 2).

Based on AK331797, P3 and P4 were designed to amplify the complete gDNA sequence of *TaPod-A2*, and were mapped on wheat chromosome 7A using a set of NT lines (data not shown). Primer sets P3 and P4 were used to produce upstream and downstream gDNA sequence, respectively, of *TaPod-A2* based on AK331797, and produced PCR products of 752 and 629 bp, respectively (Table 1). The PCR fragments amplified by P3 and P4 showed 100% sequence identify with AK331797 and had an overlap of 269 bp. The two sequences amplified from chromosome 7A were combined to create a gDNA sequence of *TaPod-A2* comprising a 5′ UTR of 92 bp, and a 3′ UTR of 1 bp, with 1182 bp in total (Table 1 and Figure 1). The ORF of *TaPod-A2* was 1089 bp, and encoded 362 AA residues with a predicted molecular weight of ∼38.9 kDa (Figure 2).

*TaPod-D1* and *TaPod-A2* lacked introns and showed the highest identities in gDNA and AA sequences, at 83.9 and 92.8%, respectively (Figure 1, 2). The ORF of *TaPod-D1* and *TaPod-A2* shared 61 SNPs and one 12-bp InDel (Figure 1); 27 SNPs gave rise to amino-acid substitutions (Figure 2). The genes *TaPod-D1* and *TaPod-A2* had an in-frame ATG codon consistent with Kozak’s rule near its 5′ end (Figure 1) (Kozak, 1996), and indicated that the trinucleotide ATG is the initiation codon of *TaPod-D1* and *TaPod-A2*.

### Characterization of Partial DNA Sequences of *TaPod-A3* and *TaPod-B1*

**FIGURE 3:**
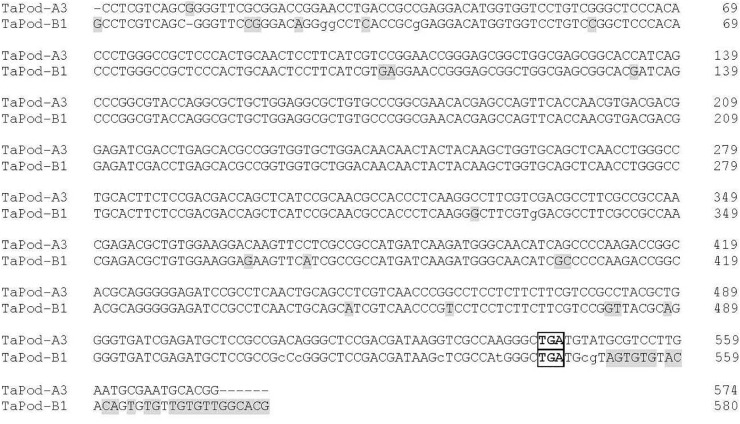
Alignment of *TaPod-A3* and *TaPod-B1*. The SNPs are *shaded*. The terminate codons are *boxed. Hyphens* indicate gaps to improve the alignment.

Primer set P5 was used to produce the downstream gDNA sequence of the *TaPod-A3* gene based on TC389044, whereas set P6 was used to produce the downstream gDNA sequence of *TaPod-B1* based on TC383617. Both of the PCR products produced by P5 and P6 showed 100% identity with the downstream sequences of TC389044 and TC383617, respectively (Figure 3). The downstream sequences of *TaPod-A3* and *TaPod-B1* showed the higher 89.6% identity (Figure 3). Nineteen SNPs and two 1-bp InDel were present in the downstream gDNA sequence of *TaPod-D1* and *TaPod-A2* (Figure 1). In this study, we failed to obtain upstream sequence of full-length gDNA sequence of *TaPod-A3* and *TaPod-B1*.

### Chromosome Localization of POD Gene Loci

Using PCR and the gene-specific primers (Table 1), all six available CS group-7 Dt lines, CS Dt7AS, CS Dt7AL, CS Dt7BS, CS Dt7BL, CS Dt7DS, and CS Dt7DL were used for the localization of POD genes to chromosome arms. PCR fragments were amplified using gene-specific primers for *TaPod-D1*, *TaPod-A2*, *TaPod-A3*, and *TaPod-B1* in five of the lines (Table 2). Among the remaining three CS NT lines, gene-specific primers failed to amplify one fragment in each. In CS Dt7DL, which lacks the short arm of chromosome 7D, no PCR product could be detected using the primers P1 and P2 for *TaPod-D1*. In CS Dt7AL, which lacks the short arm of chromosome 7A, no PCR fragment was detected using the primers P3 and P4 for *TaPod-A2*. In CS Dt7AS, which lacks the long arm of chromosome 7A; no PCR fragment could be detected using the primers P5 for *TaPod-A3*. In CS Dt7BS, which lacks the long arm of chromosome 7B, no PCR fragment could be detected using the primers P6 for *TaPod-B1* (Table 2). Overall, these results indicate that *TaPod-D1*, *TaPod-A2*, *TaPod-A3*, and *TaPod-B1* were located on chromosome 7DS, 7AS, 7AL, and 7BL, respectively.

**Table 2:** The PCR amplification *POD* genes in Chinese Spring group-7 ditelosomic lines and deletion lines.

Line	FL values	P1	P2	P3	P4	P5	P6
Amplification (+ yes, - no)	/	/	/	/	/	/	/
CS Dt7AS	/	+	+	+	+	-	+
CS Dt7AL	/	+	+	-	-	+	+
CS Dt7BS	/	+	+	+	+	+	-
CS Dt7BL	/	+	+	+	+	+	+
CS Dt7DS	/	+	+	+	+	+	+
CS Dt7DL	/	-	-	+	+	+	+
CS Del7AS-1	0.89	+	+	+	+	+	+
CS Del7AS-2	0.73	+	+	-	-	+	+
CS Del7AS-3	0.29	+	+	-	-	+	+
CS Del7AS-4	0.26	+	+	-	-	+	+
CS Del7AS-5	0.59	+	+	-	-	+	+
CS Del7AS-6	0.21	+	+	-	-	+	+
CS Del7AS-7	0.87	+	+	+	+	+	+
CS Del7AS-11	0.66	+	+	-	-	+	+
CS Del7AS-8	0.45	+	+	-	-	+	+
CS Del7AS-12	0.83	+	+	+	+	+	+
CS Del7AL-1	0.39	+	+	+	+	-	+
CS Del7AL-2	0.87	+	+	+	+	+	+
CS Del7AL-4	0.18	+	+	+	+	-	+
CS Del7AL-6	0.80	+	+	+	+	+	+
CS Del7AL-7	0.33	+	+	+	+	-	+
CS Del7AL-8	0.83	+	+	+	+	+	+
CS Del7AL-9	0.89	+	+	+	+	+	+
CS Del7AL-10	0.49	+	+	+	+	+	+
CS Del7AL-11	0.40	+	+	+	+	-	+
CS Del7AL-14	0.31	+	+	+	+	-	+
CS Del7AL-15	0.99	+	+	+	+	+	+
CS Del7AL-16	0.86	+	+	+	+	+	+
CS Del7AL-17	0.71	+	+	+	+	+	+
CS Del7AL-18	0.90	+	+	+	+	+	+
CS Del7AL-20	0.89	+	+	+	+	+	+
CS Del7AL-21	0.74	+	+	+	+	+	+
CS Del7BL-1	0.40	+	+	+	+	+	-
CS Del7BL-2	0.33	+	+	+	+	+	-
CS Del7BL-3	0.86	+	+	+	+	+	+
CS Del7BL-4	0.56	+	+	+	+	+	+
CS Del7BL-5	0.69	+	+	+	+	+	+
CS Del7BL-6	0.84	+	+	+	+	+	+
CS Del7BL-7	0.48	+	+	+	+	+	+
CS Del7BL-12	0.25	+	+	+	+	+	-
CS Del7BL-13	0.79	+	+	+	+	+	+
CS Del7DS-1	0.37	-	-	+	+	+	+
CS Del7DS-4	0.61	-	-	+	+	+	+
CS Del7DS-5	0.36	-	-	+	+	+	+


*A “-” and “+” indicate the absence and presence of PCR products, respectively, amplified with gene-specific primers for POD genes in Table 1. CS, Chinese Spring; Del, deletion lines; FL, fraction length*.

**FIGURE 4:**
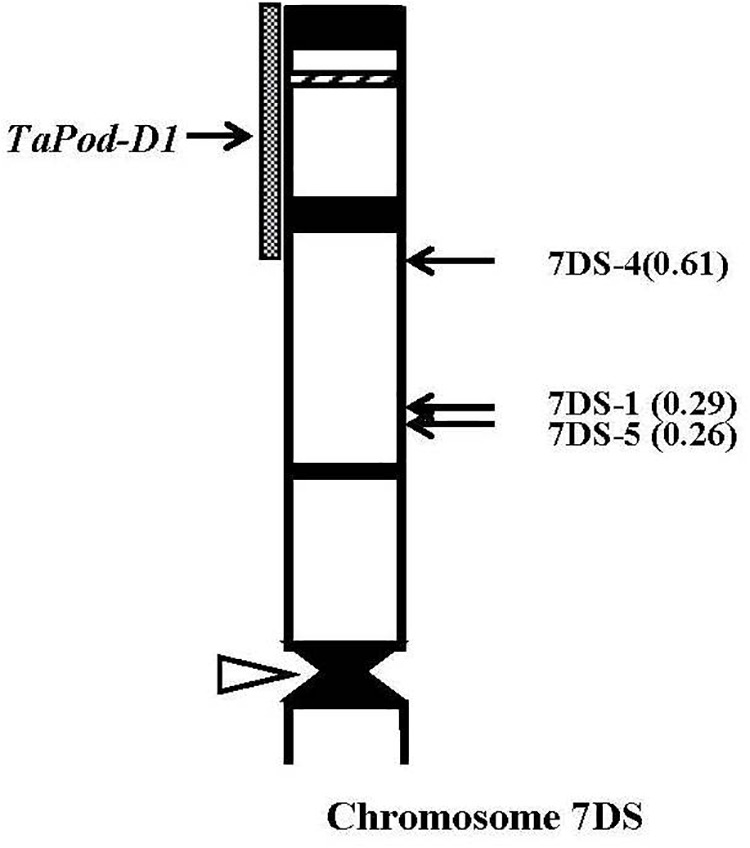
Physical map of Chinese Spring (CS) chromosome 7DS. The identification of deletion lines and fraction arm length (FL) values of breakpoints (*parentheses*) is shown on the *right*. C-banding patterns of chromosomes are taken from Gill et al. (1991). The chromosomal region (*gray hatched bar*) from FL = 0.61 to FL = 1.00 indicates the putative location of the *TaPod-D1* gene. Chromosome short arms point toward the top and the open arrow (*left*) shows the position of the centromere.

Thirty-eight group-7 Del lines were used for physical mapping of these four POD genes within each of the respective chromosome arms. The marker *TaPod-D1* appeared in all 35 Del lines; but was absent in the remaining three Del lines for 7DS with break points at Del7DS-5, FL 0.36; Del7DS-1, FL 0.37, and Del7DS-4, FL 0.61 (Table 2). These results confirmed the mapping of *TaPod-D1* in the most distal 0.39 FL portion of 7DS (Figure 4).

**FIGURE 5:**
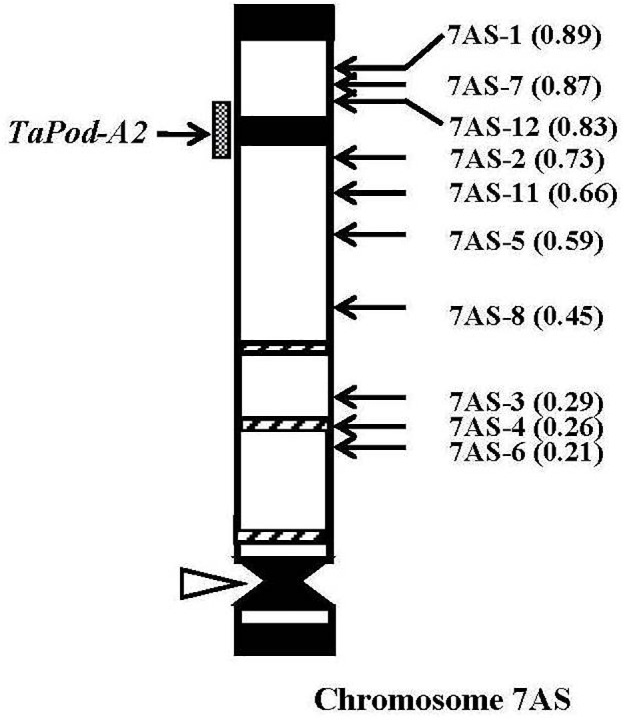
Physical map of Chinese Spring (CS) chromosome 7AS. The identification of deletion lines and fraction arm length (FL) values of breakpoints (*parentheses*) is shown on the *right*. C-banding patterns of chromosomes are taken from Gill et al. (1991). The chromosomal region (*gray hatched bar*) from FL = 0.73 to FL = 0.83 indicates the putative location of the *TaPod-A2* gene. Chromosome short arms point toward the top and the open arrow (*left*) shows the position of the centromere.

The gene-specific primers P3 and P4 for *TaPod-A2* could be used to amplify PCR products in 28 group-7 Del lines and three group-7 Del lines for 7AS; whereas no PCR product was detected in the remaining seven Del lines for 7AS (Del7AS-6, FL 0.21; Del7AS-4, FL 0.26; Del7AS-3, FL 0.29; Del7AS-8, FL 0.45; Del7AS-5, FL 0.59; Del7AS-11, FL 0.66; and Del7AS-2, FL 0.73) (Table 2), which confirmed the location of *TaPod-A2* between breakpoint FL 0.73 (Del7AS-2) and FL 0.83 (Del7AS-12) of the euploid chromosome of CS (Figure 5).

**FIGURE 6:**
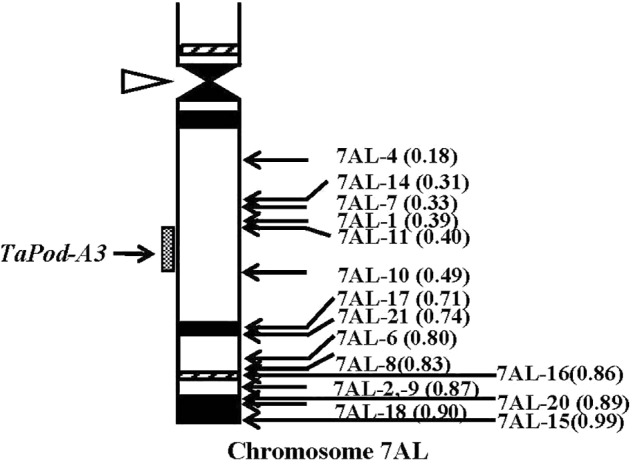
Physical map of Chinese Spring (CS) chromosome 7AL. The identification of deletion lines and fraction arm length (FL) values of breakpoints (*parentheses*) is shown on the *right*. C-banding patterns of chromosomes are taken from Gill et al. (1991). The chromosomal region (*gray hatched bar*) from FL = 0.40 to FL = 0.49 indicates the putative location of the *TaPod-A3* gene. Chromosome long arms point toward the bottom and the open arrow (*left*) shows the position of the centromere.

The primers P5 for *TaPod-A3* could amplify PCR products in 22 group-7 Del lines, and 11 group-7 Del lines for 7AL; whereas no PCR product was detected in the remaining five Del lines for 7AL (Del7AL-4, FL 0.18; Del7AL-14, FL 0.31; Del7AL-7, FL 0.33; Del7AL-1, FL 0.39, and Del7AL-11, FL 0.40) (Table 2), indicating that *TaPod-A3* is located between breakpoint FL 0.40 (Del7DL-11) and FL 0.49 (Del7DL-10) of the euploid chromosome of CS (Figure 6).

**FIGURE 7:**
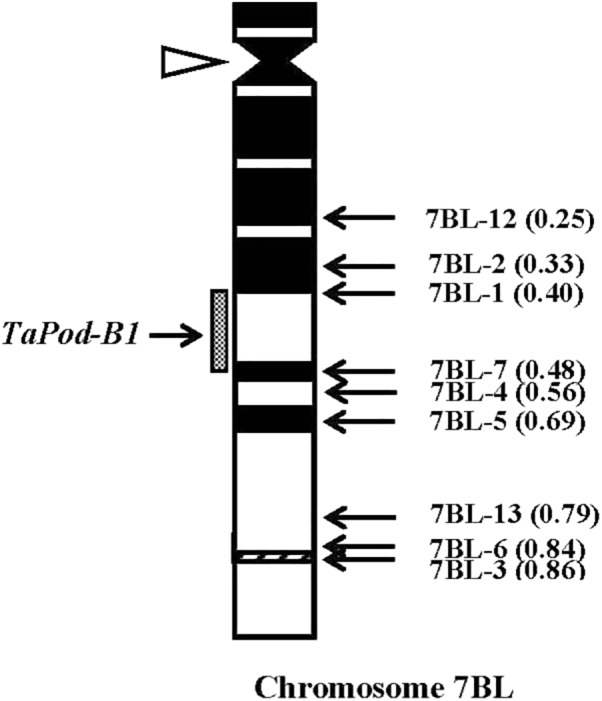
Physical map of Chinese Spring (CS) chromosome 7BL. The identification of deletion lines and fraction arm length (FL) values of breakpoints (*parentheses*) is shown on the *right*. C-banding patterns of chromosomes are taken from Gill et al. (1991). The chromosomal region (*gray hatched bar*) from FL = 0.40 to FL = 0.48 indicates the putative location of the *TaPod-B1* gene. Chromosome long arms point toward the bottom and the open arrow (*left*) shows the position of the centromere.

The primers P6 for *TaPod-B1* could amplify PCR products in 29 group-7 Del lines, and six Del lines for 7BL; but no PCR product was detected in the remaining three Del lines for 7BL (Del7BL-12, FL 0.25; Del7BL-2, FL 0.33, and Del7BL-1, FL 0.40) (Table 2), indicating that *TaPod-B1* is located between breakpoint FL 0.40 (Del7BL-1) and FL 0.48 (Del7BL-7) of the euploid chromosome of CS (Figure 7).

### Allelic Variants at the *TaPod-D1* Locus

**FIGURE 8:**
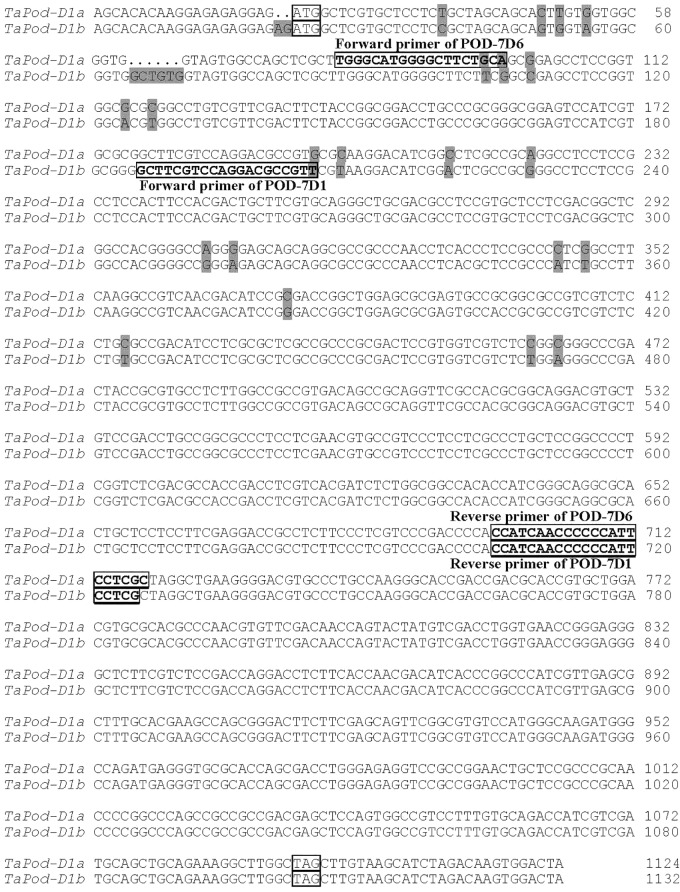
Alignment of alleles *TaPod-D1a* and *TaPod-D1b* mapped on wheat chromosome 7D. The initiation and termination codons are *boxed*; the SNPs are *shadowed*; the forward and reverse primers of POD-7D6 and POD-7D1 are *boxed* and *bold*.

The gDNA sequence of *TaPod-D1* was 1124 bp, and contains an exon of 1077 bp, a 5′ UTR of 20 bp, and a 3′ UTR of 27 bp. Two *TaPod-D1* alleles were detected from varieties with higher and lower POD activities, and designated *TaPod*-*D1a* and *TaPod*-*D1b*, respectively (Figure 8). One Indel and 21 SNPs were found between two alleles in the exon of *TaPod*-*D1*, with an Indel (GCTGTG) between the 42nd and 43rd base of *TaPod*-*D1a*, the other SNPs are illustrated in Figure 8. The deduced protein sequences of these two *TaPod-D1* alleles, *TaPod*-*D1a* and *TaPod*-*D1b*, contained 357 and 359 AA residues with deduced molecular masses of 38.8 and 39.1 kDa, respectively, and shared 98.6% similarity. Two new AA residues and five missense mutations were found between two alleles in the AA residues of *TaPod-D1*, with the two new AA (Alanine, A; Valine, V) between the 15th and 16th AA of *TaPod-D1a*. The other mutations are shown in Supplementary Figure S2.

### Development of the *TaPod-D1* STS Markers for POD Activity

Two functional markers, POD-7D6 and POD-7D1, were developed based on the three mutations at the 76th (from G to T), 78th (from A to G) and 177th (from G to T) bases of *TaPod-D1a* (Figure 8 and Table 1). POD-7D6 can amplify a PCR fragment of 640 bp in the *TaPod-D1a* genotypes with lower POD activity; whereas no product is produced in the *TaPod-D1b* genotypes with higher POD activity (Supplementary Figure S3a). In contrast, POD-7D1 amplifies a product of 540 bp in the *TaPod-D1b* genotypes with higher POD activity; whereas no product is produced in the *TaPod-D1a* genotypes with lower POD activity (Supplementary Figure S3b). The two complementary dominant functional markers POD-7D6 and POD-7D1 were physically mapped on 7DS by amplifying NT and three Del lines of chromosome 7DS (CS Del7DS) (Supplementary Figure S4).

The POD activities of 224 wheat varieties were associated with these two functional markers POD-7D6 and POD-7D1. Of the 224 varieties, 115 varieties had *TaPod*-*D1a* and 109 had *TaPod*-*D1b* (Table 3). The ANOVA indicated that the average POD activities of the 115 varieties with *TaPod*-*D1a* expressed 654.6, 705.7, and 675.7 U min^-1^ g^-1^ for Regions YHRVWWR and NWWR, and the combined data, respectively, and were significantly lower than the 109 varieties with *TaPod*-*D1b*, which expressed 689.3, 760.1, and 709.0 U min^-1^ g^-1^ for Regions YHRVWWR and NWWR, and the combined data, respectively (*P* < 0.01) (Table 3).

**Table 3:** Allelic variants at the *TaPod-A2* locus in wheat varieties and POD activities with the markers POD-7D1 and POD-7D6.

Region^a^	Marker	Genotype^b^	No. accessions	Mean POD activity ^c^	Range
YHRVWWR	POD-7D1	*TaPod-D1b*	83	689.3a	431.3–954.8
	POD-7D6	*TaPod-D1a*	64	654.6b	461.4–874.2
NWWR	POD-7D1	*TaPod-D1b*	32	757.8a	526.4–1111.8
	POD-7D6	*TaPod-D1a*	45	706.7b	516.6–850.3
Total	POD-7D1	*TaPod-D1b*	115	708.1a	461.4–874.2
	POD-7D6	*TaPod-D1a*	109	676.1b	431.3–1111.8


*^a^YHRVWWR, Yellow and Huai River Valley Winter Wheat Region; NWWR, Northern China Plain Winter Wheat Region. ^*b*^The genotypes were determined by the 540- and 640-bp polymerase chain reaction fragments amplified with POD-7D1 and POD-D6, respectively. ^*c*^Different letters following the mean POD activities indicate highly significant differences between the two genotypic groups (*P* < 0.01; Fisher’s protected LSD). The POD activity (U min^-*1*^ g^-*1*^) of whole wheat meal was from Wei et al. (2015)*.

## Discussion

### Comparison of Structures of *TaPod-D1*, *TaPod-A2*, and Other Plant *POD* Genes

A striking feature of the nucleotide sequence of POD cDNA in cereals is the GC-richness (∼70.0% G/C)(Theilade and Rasmussen, 1992; Passardi et al., 2004b; Alexandrov et al., 2009). Here, the complete gDNA sequences of *TaPod-A2* and *TaPod-D1* were characterized *in silico* in combination with PCR amplification. The overall G/C content of common wheat *TaPod-A2* and *TaPod-D1* cDNA sequences is 70.0 and 69.5%, respectively. Similarly, the cDNA sequences of rice *OsPrx23* (BN000552), barley *HvPrx6* (M83671), and maize POD genes (EU962973) have 69.9, 69.0, and 72.1% G/C contents, respectively. Shi and Jarvis (2006) documented that the full-length gDNA sequence of genes that are GC-rich were difficult to isolate with the rapid amplification of cDNA ends (RACE) technique. We found that the G/C content of upstream sequences of wheat POD genes was significantly higher than the G/C content of downstream sequences (∼73.0 vs. ∼66.0%), which may be one of the reasons that we failed to obtain upstream sequence of *TaPod-A3* and *TaPod-B1* by *in silico* analysis in combination with PCR amplification.

In sequence alignments of POD genes, the gDNA of *TaPod-A2* and *TaPod-D1* exhibited, respectively, 60.2 and 62.1% sequence identities with *HvPrx6*, 73.9 and 73.6% with *OsPrx23*, and 71.8 and 73.9% maize POD gene (EU962973); whereas *TaPod-A2* and *TaPod-D1* cDNA showed 79.3 and 79.2% sequence identities with *HvPrx6*, 75.4 and 74.6% with *OsPrx23*, and 75.8 and 6.1% with maize POD gene (EU962973). The homologies of gDNA and cDNA sequences among the POD genes of maize, rice, and common wheat ranged from 54.0 to 94.1% (data not shown), with the lowest homology among gDNA sequences due to discrepancies among introns with much lower levels of conserved sequence (Theilade and Rasmussen, 1992; Passardi et al., 2004b; Alexandrov et al., 2009).

The deduced AA sequences of *TaPod-A2* and *TaPod-D1* exhibited 65.6–69.2% identities with *HvPrx6*, *OsPrx23*, and maize POD gene (EU962973), respectively (Figure 2). All of the POD genes included three conserved domains of class III PODs, six cysteines involved in disulfide bridges, and four residues involved in a salt bridge (Figure 2) (Theilade and Rasmussen, 1992; Passardi et al., 2004b; Delannoy et al., 2006; Alexandrov et al., 2009; Marchler-Bauer et al., 2009). Among the three highly conserved domains of class III proxidases, the first and third domains are the distal and proximal heme-binding domains, respectively, whereas the second domain is the central conserved domain and has no known function in proxidase activity (Hiraga et al., 2001; Delannoy et al., 2006; Shigeto and Tsutsumi, 2016).

Alignment of the exons of POD genes indicated that *TaPod-A2*, *TaPod-D1*, *HvPrx6*, *OsPrx23*, and maize POD genes (EU962973) have full-length 1086, 1074, 1077, and 1068 bp cDNA sequences, respectively. Sequence alignments of POD genes of rice (*OsPrx23*, *OsPrx76*, *OsPrx84*, *OsPrx87*, *OsPrx89*, and *OsPrx91*), maize (EU962973), barley (*HvPrx5* and *HvPrx6*), and common wheat (*TaPod-A2* and *TaPod-D1*) indicated that the exon–intron structures of POD genes are diversified. Until now, five types of exon–intron structures of POD genes in cereals were found, viz., five-exon/four-intron (*OsPrx91*), four-exon/three-intron (*OsPrx89*), three-exon/two-intron (*OsPrx87*), two-exon/one-intron (*OsPrx84*, *HvPrx6*), and one-exon/zero-intron structures (*TaPod-A2*, *TaPod-D1*, *HvPrx5*, and *OsPrx76*) (Theilade and Rasmussen, 1992; Passardi et al., 2004b; Alexandrov et al., 2009). In particular, the one-exon/zero-intron structures are found in most crop species. The results indicated that there were relatively large differences in exon–intron structures among different cereals. In the same cereal species, especially rice, the exon–intron structure of POD genes was also different.

### Verification of the Location of *POD* Genes

Kobrehel and Feillet (1975) found that POD isozymes a and d were located on chromosomes 7D and 7A, respectively. In this study, we mapped *TaPod-A2* to 7AS and *TaPod-D1* to 7DS using NT, Dt and Del lines of CS. Therefore, *TaPod-A2* and *TaPod-D1* may code POD isozymes d and a, respectively. This study verifies the previous mapping data on the existence of *TaPod-A2* and *TaPod-D1* loci on 7AS and 7DS. Our study further localized the *TaPod-A2*, *TaPod-A3*, *TaPod-B1*, and *TaPod-D1* genes on the terminal regions of 7AS, 7AL, 7BL, and 7DS, respectively. Based on sequence analysis, gDNA of *TaPod-A2* and *TaPod-D1* had 56.5 and 55.7% sequence identities with *OsPrx76* gene (GenBank accession number: BN000605) in *Oryza sativa*, respectively (Passardi et al., 2004b). The rice PODs were earlier reported on rice chromosome 6, which is syntenous to wheat group-7 chromosomes (Sorrells et al., 2003; Passardi et al., 2004b). These results would agree with the synteny established between chromosomes of rice and wheat (Sorrells et al., 2003).

Here, we found that *TaPod-A2* and *TaPod-D1* were located at the similar breakpoint region of 7AS (between breakpoint FL 0.73 and FL 0.83) and 7DS (between breakpoint FL 0.61 and FL 1.00), respectively. Further, *TaPod-A3* and *TaPod-B1* were located at the similar breakpoint region of 7AL (between breakpoint FL 0.40 and FL 0.49) and 7BL (between breakpoint FL 0.40 and FL 0.48), respectively. Sequence analysis of gDNA and cDNA obtained from various cloning experiments and this study suggests that there are more than four copies of the POD gene in hexaploid wheat. Interestingly, in this study, we found that POD genes were located on either on 7AS (*TaPod-A2*) or 7DS (*TaPod-D1*), but not on 7BS. In a previous work, Kobrehel and Feillet (1975) also localized two POD isozymes a and d on 7AS and 7DS in wheat, respectively, and failed to find any POD isozymes on chromosomes 7B. This may be due to homoeologies within groups 4 and 7 and are affected by several rearrangements known to have occurred on and 7BS (Liu et al., 1992; Nelson et al., 1995).

### Allelic Variants of *TaPod-D1* and POD Activity

Transmembrane proteins (TMPs) are a type of integral membrane and polytopic proteins, which are comprised of a region of AA with a largely hydrophobic character. These proteins have a gateway function that allows specific substances to be transported across the biological membrane. The hydrophobic group of TMP is the primary factor determining the functional characteristics of TMP (Liu et al., 2002). In this study, compared to *TaPod-D1a*, *TaPod-D1b* has two more hydrophobic AAs (Alanine and Valine) residues between the 15th and 16th residues in the protein sequence of *TaPod-D1a*, which would likely lead to a new conformation in this C-terminal portion of the protein. The number of hydrophobic AAs was not changed due to the other five variant AA residues (Supplementary Figure S2). These mutations are located near the C-terminal domain of the conserved 5′ end of the POD gene, which is an important domain in maintaining the function of POD genes (Hiraga et al., 2001; Passardi et al., 2004b; Marchler-Bauer et al., 2009). These polymorphisms could be the reason for a higher expression of the POD gene in the genotype *TaPod-D1b* attributable to a higher transmembrane transport capacity.

## Conclusion

The complete gDNA sequence of *TaPod-A2* and *TaPod-D1*, and the partial gDNA sequence of two additional POD genes (*TaPod-A3* and *TaPod-B1*) were characterized. *TaPod-A2* and *TaPod-D1* were found to be physically located on 0.73–0.83 and on the most distal 0.39 fraction arm length (FL) of 7AS and 7DS; whereas, *TaPod-A3* and *TaPod-B1* were assigned to the 0.40–0.49 and 0.40–0.48 FL of 7AL and 7BL, respectively. Two functional markers POD-7D1 and POD-7D6 were developed, amplifying 540- and 640-bp, fragments in varieties with higher and lower POD activities, respectively. The ANOVA indicated the average POD activities of these varieties with *TaPod*-*D1a* were significantly lower than these varieties with *TaPod*-*D1b* (*P* < 0.01). This study provides useful information about the molecular structure of the POD genes, effective gene-specific markers, and contributes a valuable resource for quality improvement in wheat breeding programs.

## Author Contributions

HG performed the experiments and wrote the manuscript. JS and JW performed a portion of the experiments. EF and CM designed the experiments and contributed to writing of the manuscript.

## Conflict of Interest Statement

The authors declare that the research was conducted in the absence of any commercial or financial relationships that could be construed as a potential conflict of interest.

## Footnotes

1 https://www.k-state.edu/wgrc/genetic_resources/deletion_lines/group_7.html (accessed February 16, 2019).

2 http://compbio.dfci.harvard.edu/tgi/cgibin/tgi/blast/index.cgi is no longer active (accessed February 12, 2014).

3 http://www.premierbiosoft.com/ (accessed February 16, 2019).

4 http://www.lynnon.com (accessed February 16, 2019).

5 http://www.ncbi.nlm.nih.gov/Structure/cdd/wrpsb.cgi (accessed February 16, 2019).

6 http://compbio.dfci.harvard.edu/tgi/cgibin/tgi/blast/index.cgi is no longer active (accessed February 12, 2014).
