# Supplementary material for: Physical Mapping of Peroxidase Genes and Development of Functional Markers for TaPod-D1 on Bread Wheat Chromosome 7D
Source: Front Plant Sci. 2019 Apr 24;10:523. doi: 10.3389/fpls.2019.00523 (PMC6491870; doi:10.3389/fpls.2019.00523)
Supplement: Supplementary file 1 [file Table_1.doc]

**Appendix**

M 1 2 3 4 5 6 7 8 9 10 11 12 13 14 15 16 17 18 19 20 21 M


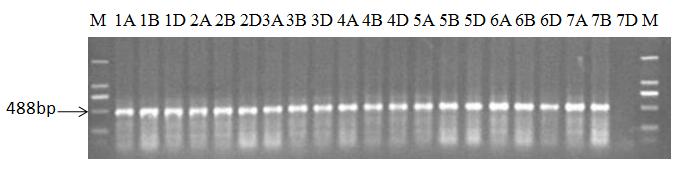


**a**


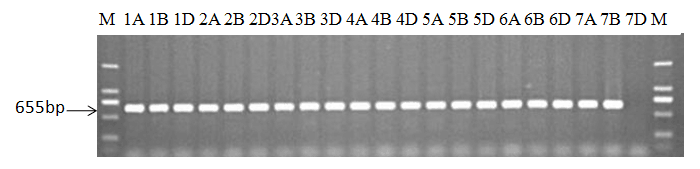


M 1 2 3 4 5 6 7 8 9 10 11 12 13 14 15 16 17 18 19 20 21 M

**b**

**Fig. S1.** PCR amplification of CS NT lines with primer combinations P1 (a) and P2 (b). No PCR fragment was detected in N7D-T7A, whereas the amplification was generated in other nullisomic-tetrasomics, showing that *TaPod-D1* was mapped on 7D. M DL2000; 1 N1A-T1B; 2 N1B-T1D; 3 N1D-T1A; 4 N2A-T2D; 5 N2B-T2A; 6 N2D-T2B; 7 N3A-T3B; 8 N3B-T3D; 9 N3D-T3A; 10 N4A-T4B; 11 N4B-T4D; 12 N4D-T4A; 13 N5A-T5B; 14 N5B-T5D; 15 N5D-T5A; 16 N6A-T6B; 17 N6B-T6D; 18 N6D-T6A; 19 N7A-T7B; 20 N7B-T7D; 21 N7D-T7A.

TaPod-D1a MARAPLLAALVVAV..VVASSLGHGASAAEPPVARGLSFDFYRRTCPRAESIVRGFVQDAVRKDIGLAAG 68

TaPod-D1b MARAPPLAAVVVAVAVVVASSLGHGASSAEPPVARGLSFDFYRRTCPRAESIVRGFVQDAVRKDIGLAAG 70

TaPod-D1a LLRLHFHDCFVQGCDZSVLLDGSATGPGEQQAPPNLTLRPSAFKAVNDIRDRLERECRGAVVSCADILAL 138

TaPod-D1b LLRLHFHDCFVQGCDZSVLLDGSATGPGEQQAPPNLTLRPSAFKAVNDIRDRLERECHRAVVSCADILAL 140

TaPod-D1a AARDSVVVSGGPDYRVPLGRRDSRRFATRQDVLSDLPAPSSNVPSLLALLRPLGLDATDLVTISGGHTIG 208

TaPod-D1b AARDSVVVSGGPDYRVPLGRRDSRRFATRQDVLSDLPAPSSNVPSLLALLRPLGLDATDLVTISGGHTIG 210

TaPod-D1a QAHCSSFEDRLFPRPDPTINPPFLARLKGTCPAKGTDRRTVLDVRTPNVFDNQYYVDLVNREGLFVSDQD 278

TaPod-D1b QAHCSSFEDRLFPRPDPTINPPFLARLKGTCPAKGTDRRTVLDVRTPNVFDNQYYVDLVNREGLFVSDQD 280

TaPod-D1a LFTNDITRPIVERFARSQRDFFEQFGVSMGKMGQMRVRTSDLGEVRRNCSARNPGPAAADELQWPSFVQT 348

TaPod-D1b LFTNDITRPIVERFARSQRDFFEQFGVSMGKMGQMRVRTSDLGEVRRNCSARNPGPAAADELQWPSFVQT 350

TaPod-D1a IVDAAAERL 357

TaPod-D1b IVDAAAERL 359

**Fig. S2.** Amino acid sequence alignment of wheat *TaPod-D1b* and *TaPod-D1b*. The different amino acids in the peptide sequences encoded by *TaPod-D1a* and *TaPod-D1b* are *shadowed*. Among these mutation and insertion/deletion sites, the hydrophobic amino acids are *boxed.*

**640 bp**


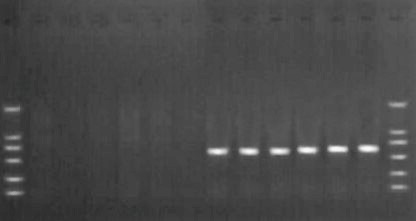

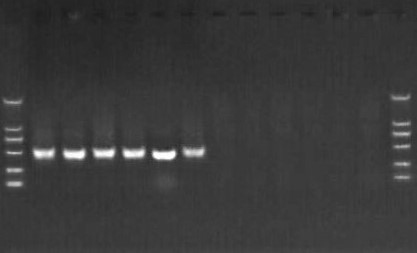


**M 1 2 3 4 5 6 7 8 9 10 11 12 M**

**a**

**b**

**540 bp**

**Fig. S3.** Polymorphism of PCR fragments amplified by POD-7D6 (a) and its complementary marker POD-7D1 (b) in six varieties with lower POD activity and six with higher POD activity. M: DNA ladder DL2000; 1 CA1133; 2 CA1135; 3 Lankao 24; 4 Zhong 892; 5 Zhongmai 175; 6 Zhou 8425B; 7 Lumai 6; 8 CA1090; 9 Shan 715; 10 Yumai 21; 11 Zhoumai 16; 12 Shi 4185.


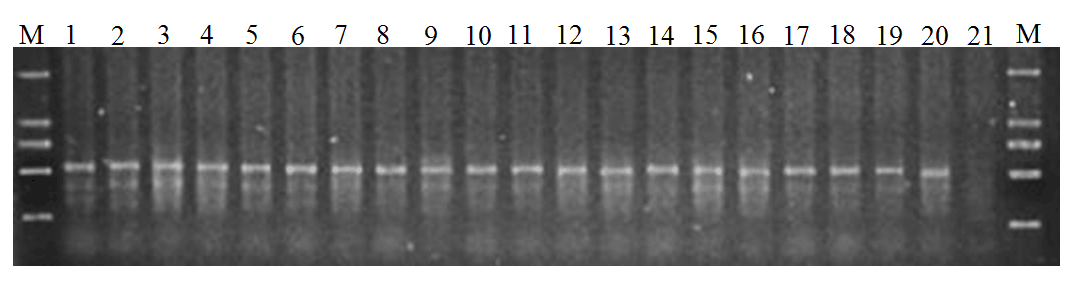


**Fig. S4.** PCR amplification of CS nullisomic-tetrasomic lines with primer set POD-7D1. No expected PCR fragment was detected in N7D-T7A, whereas the expected PCR fragment was observed in other corresponding nulli-tetrasomics, indicating that the gene-specific marker was localized on wheat chromosome 7D. M DNA ladder DL2000; 1 Chinese Spring nullisomic 1A–tetrasomic 1D (N1A-T1D); 2 N1B-T1D; 3 N1D-T1B; 4 N2A-T2B; 5 N2B-T2A; 6 N2D-T2B; 7 N3A-T3D; 8 N3B-T3D; 9 N3D-T3A; 10 N4A-T4B; 11 Dt4BS; 12 N4D-T4B; 13 N5A-T5B; 14 N5B-T5A; 15 N5D-T5B; 16 N6A-T6B; 17 N6B-T6D; 18 N6D-T6B; 19 N7A-T7B; 20 N7B-T7D; 21 N7D-T7A.
